# Impact of Melatonin Supplementation on Sports Performance and Circulating Biomarkers in Highly Trained Athletes: A Systematic Review of Randomized Controlled Trials

**DOI:** 10.3390/nu16071011

**Published:** 2024-03-30

**Authors:** Ana M. Celorrio San Miguel, Enrique Roche, María Herranz-López, Marta Celorrio San Miguel, Juan Mielgo-Ayuso, Diego Fernández-Lázaro

**Affiliations:** 1 Department of Chemistry, Polytechnic Secondary Education High School, 42004 Soria, Spain; amcelorrio@educa.jcyl.es; 2 Doctoral School, University of León, Campus de Vegazana, 24071 Leon, Spain; 3 Department of Applied Biology-Nutrition, Institute of Bioengineering, University Miguel Hernandez, 03202 Elche, Spain; eroche@umh.es; 4 Alicante Institute for Health and Biomedical Research (ISABIAL), 03010 Alicante, Spain; 5 CIBER Physiopathology of Obesity and Nutrition (CIBEROBN), Carlos III Health Institute (ISCIII), 28029 Madrid, Spain; 6 Research Group “Nutrition and Physical Activity”, Spanish Nutrition Society “SEÑ”, 28010 Madrid, Spain; jfmielgo@ubu.es; 7 Institute of Research, Development, and Innovation in Healthcare Biotechnology of Elche (IDiBE), Miguel Hernández University (UMH), 03202 Elche, Spain; mherranz@umh.es; 8 Emergency Department, Línea de la Concepción Hospital, C. Gabriel Miró, 108, 11300 La Línea de la Concepción, Spain; marta.celorrio.sspa@juntadeandalucia.es; 9 Department of Health Sciences, Faculty of Health Sciences, University of Burgos, 09001 Burgos, Spain; 10 Department of Cellular Biology, Genetics, Histology and Pharmacology, Faculty of Health Sciences, University of Valladolid, Campus of Soria, 42004 Soria, Spain; 11 Neurobiology Research Group, Faculty of Medicine, University of Valladolid, 47005 Valladolid, Spain

**Keywords:** melatonin, sports supplementation, sports performance, safety, oxidative stress, antioxidant, inflammatory response, health biomarkers, highly trained athletes

## Abstract

Melatonin (N-acetyl-5 methoxytryptamine) is an indolic neurohormone that modulates a variety of physiological functions due to its antioxidant, anti-inflammatory, and immunoregulatory properties. Therefore, the purpose of this study was to critically review the effects of melatonin supplementation in sports performance and circulating biomarkers related to the health status of highly trained athletes. Data were obtained by performing searches in the following three bibliography databases: Web of Science, PubMed, and Scopus. The terms used were “Highly Trained Athletes”, “Melatonin”, and “Sports Performance”, “Health Biomarkers” using “Humans” as a filter. The search update was carried out in February 2024 from original articles published with a controlled trial design. The PRISMA rules, the modified McMaster critical review form for quantitative studies, the PEDro scale, and the Cochrane risk of bias were applied. According to the inclusion and exclusion criteria, 21 articles were selected out of 294 references. The dose of melatonin supplemented in the trials ranged between 5 mg to 100 mg administered before or after exercise. The outcomes showed improvements in antioxidant status and inflammatory response and reversed liver damage and muscle damage. Moderate effects on modulating glycemia, total cholesterol, triglycerides, and creatinine were reported. Promising data were found regarding the potential benefits of melatonin in hematological biomarkers, hormonal responses, and sports performance. Therefore, the true efficiency of melatonin to directly improve sports performance remains to be assessed. Nevertheless, an indirect effect of melatonin supplementation in sports performance could be evaluated through improvements in health biomarkers.

## 1. Introduction

In humans, the neurohormone melatonin (N-acetyl-5 methoxy-tryptamine) is synthesized by the pineal gland and secreted into the central nervous system internal milieu [1]. The pineal gland is a neuroendocrine photosensor that receives light stimuli transforming it into humoral signals, although other different activities have been documented [2]. In this context, melatonin modulates a variety of physiological functions, such as the regulation of the sleep/wake cycle and circadian rhythms as the most important actions. In addition, melatonin exerts additional functions such as neuro- and cardioprotective agents, anti-tumor, anti-aging, and protecting the structural integrity and bioenergetic activity of mitochondria against oxidative damage. For these attributed properties, melatonin is classified as an antioxidant, anti-inflammatory, immunomodulator, and antitumoral neurohormone [3,4]. This suggests that melatonin would play a key role in the recovery from the disruptive processes derived from high-intensity exercises [5]. This type of action results in alterations in the homeostasis of metabolic, hormonal, neuro-muscular, and immunological systems that occur as a result of local and systemic inflammatory responses to strenuous exercise [6,7]. This particular situation can cause decreased sports performance, fatigue, and overtraining [8].

Highly demanding training exponentially increases physical and biochemical demands, particularly in the skeletal muscle and the liver [9]. This situation induces extra-metabolic needs that cause an increase in nutrient consumption [10], and alterations in the physical, thermic, and mechanical conditions of these tissues, resulting in the accumulation of potentially harmful metabolic molecules such as free radicals and stress messengers [11]. This condition promotes the activation and attraction of inflammatory cells to muscle tissue, altering redox homeostasis. It has to be emphasized that the stress caused by intense training could progress to chronic and/or systemic inflammation if poor rest and inadequate recovery are wrongly scheduled post-exercise [12]. Additionally, the athlete’s immune system can become functionally depressed as a result of extreme physical stress, as described in elite/professional sports [13,14]. In this context, sleep is a restorative element that improves performance as a result of optimal recovery of athletes [15]. Lack of sleep induces an increase in catabolic hormones and a reduction in anabolic hormones, leading to impaired protein synthesis and tissue repair [16]. This hinders adaptations to training, recovery processes, and subsequent sports performance [17].

Altogether, variations occur in the set of circulating health biomarkers that are conditioned by the type and intensity of exercise, the physical condition of the athlete, the nutritional status of the individual, and external environmental factors [18]. To maintain biomarkers in a healthy state for optimal performance, specific nutritional actions and dietary supplementation interventions can be undertaken, especially during intense training in the preparation phase or during very demanding competition moments. With this in mind, intense days of training and competition require extra recovery, being necessary the intake of nutritional supplements that help improve metabolic and physiological recovery, particularly in muscle tissue [19,20]. For this reason, multiple studies [20,21,22] have been carried out to demonstrate the effects of different antioxidant substances and recovery products on functional and structural damage.

One of the molecules that has shown a key role after very demanding exercise is melatonin. This is due to its pleiotropic bioactions by interaction with membrane receptors that trigger signaling pathways activating different enzymes and transcription factors in different tissues. Therefore, in addition to circadian rhythm regulation, melatonin exerts antioxidant, anti-inflammatory, immunostimulant, cardioprotective, antidiabetic, anti-obesity, neuroprotective, anti-aging, and anti-neoplastic actions [4,23]. Inflammation resulting from extreme exercise actions generates elevated secretion of inflammatory cytokines and chemokines [24]. Melatonin works as an anti-inflammatory agent leading to a reduction in the production of proinflammatory cytokines such as TNF-α (tumor necrosis factor-α), IL-1β (interleukin-1β), IL-6 and IL-8, and an increase in the level of the anti-inflammatory cytokines IL-4 and IL-10 [25]. In addition, melatonin is considered one of the most potent antioxidants due to its ability to directly scavenge free radicals [26] and indirectly enhance the expression of enzymes involved in redox metabolism [27]. The chemical structure of melatonin and its high redox potential allows it to easily give electrons, favoring the reduction and elimination of free radicals and reactive oxygen and nitrogen species (RONS) in many body systems [28]. Regarding the indirect antioxidant mechanisms, melatonin inhibits pro-oxidative enzymes [29] and increases the expression and activity of antioxidant enzymes, such as catalase (CAT), superoxide dismutases (SOD1, SOD2), glutathione peroxidase and reductase (GPx, GRd), peroxiredoxin (PRs), and glutathione-γ-glutamylcysteine synthetase; the last four enzymes are responsible for regulating the redox cycle of glutathione (GSH), the key antioxidant in humans [30]. 

The effect of melatonin on mitigation of damage at the physiological, biochemical, and metabolic levels following demanding exercise is currently unclear. This can condition sports performance. Therefore, the physiological effects of melatonin supplementation could be advantageous for restoration of certain health biomarkers and recovery after certain demanding physical actions. However, a recent systematic review [31] did not clarify whether there is an effect of melatonin administration on physical performance. Melatonin did not improve strength, power, speed, and short-duration continuous exercise in trained athletes. However, an improvement in balance and performance of long-term continuous exercise was reported in non-athletes [31]. For this reason, this systematic review will aim to identify, evaluate, analyze, and summarize the findings of relevant original studies regarding the effects of melatonin administration on several parameters, including: circulating biomarkers (blood glucose, lipid metabolism, kidney function, liver function, hormone response, inflammatory response, and muscle damage), antioxidant status (antioxidant enzymes, oxidative stress biomarkers, antioxidant function and glutathione homeostasis), perceptual and cognitive response, physiological biomarkers, melatonin bioavailability, adverse effects of melatonin, and sports performance (long-term continuous exercise, aerobic capacity, strength, and power) in highly trained athletes.

## 2. Materials and Methods

### 2.1. Protocol and Registration

This systematic review has been prepared following the Preferred Reporting Items for Systematic Reviews and Meta-Analyses (PRISMA) guidelines [32]. The current protocol was registered in the International Prospective Register of Systematic Reviews (PROSPERO) database (#CRD 42024504290). 

### 2.2. Literature Search Strategy

A systematic literature search was conducted using the following databases: Medline (PubMed), Scopus, and Web of Science (WOS) including all the results published between January 2006 and February 2024. The search strategy was restricted to studies published in English and Spanish, excluding the manuscripts published in other languages. The search was conducted using keywords connected by Boolean connectors. The search strategy used in databases included terms related to melatonin and the different outcomes of circulating parameters that change according to health status and exercise practice and recovery, as well as a combination of these using the Medical Subject Headings (MeSH) index and Boolean operators: (“Melatonin” OR “N-acetyl-5-methoxytryptamine” AND (“Trained Athletes” OR “Professional Athletes” OR “Sports” OR “Athletic Performance” OR “Sports performance” OR “exercise/physiology” OR “muscle, skeletal” OR “Physical Fitness” OR “Cardiorespiratory Fitness”) AND (“Adaptations” OR “Markers” OR “Effects” OR “Analysis” OR “Biomarkers” OR “Indicators” OR “Health Status” OR “Activity” OR “Pathways) NOT (“Syndrome” OR “Disease” or “Therapy” OR “Wounds and injuries”). Other studies were obtained through a “snowball” search using the references included in the eligible publications for full-text review and use of the ResearchGate platform to identify possible articles not included in the databases previously described in our study.

### 2.3. Eligibility Criteria

The research question for this systematic review was: “Could Melatonin supplementation improve sports performance and/or health status in highly trained athletes?” To answer this question, inclusion criteria were established based on the PICOS [33] criteria (Table 1).

### 2.4. Study Selection

Two authors (A.M.C. and D.F.-L.) independently reviewed all the titles and abstracts obtained from the search strategy in the three databases used. These reviewers excluded all the manuscripts that did not align with the objectives of this review. In a subsequent phase, the same authors thoroughly revised the full texts and checked if the manuscript fulfilled all the inclusion criteria. In the first (title and abstract screening) and second phase (full-text review), a third reviewer (E.R.) reviewed and solved any discrepancies. Lastly, manuscripts that include only a probabilistic magnitude-based inference were removed. Also, to carry out the selection of studies, we used the Covidence systematic review software (3.0-2024) for the selection and recording of decisions.

### 2.5. Data Extraction

Two authors (A.M.C. and D.F.-L.) extracted data independently using the Covidence systematic review software. Any discrepancy was resolved through discussion, and a third author (E.R.) was consulted if consensus was required. The information on each study included in the systematic review consisted of the following:

- Study: name of the first author, year of publication, country in which the study was conducted;
- Sample: The number of participants and the main characteristics related to their sports practice: trained, professional, or competitive athletes, initial sample size, age, sex, anthropometric and physical characteristics, withdrawals from the study, and final group sample size;
- Study design: types of randomized clinical trial;
- Intervention: description of each intervention, dose, pharmaceutical form, composition, timing and hour of supplementation, duration, and washout periods;
- Outcomes: all the outcomes assessed related to sports performance and/or health status (through clinical laboratory analytical biomarkers);
- Results: studies that specify the results where statistical differences were found between the experimental conditions (melatonin supplementation group and the placebo group).

### 2.6. Methodological Quality Assessment

The methodological evaluation of the selected trials was carried out using the Physiotherapy Evidence Database (PEDro) scales [34], and the critical review form for quantitative studies developed by the McMaster University Occupational Therapy Evidence-Based Practice Research Group (McMaster) as a critical appraisal tool [35]. Both guides [34,35] are appropriate for the evaluation of randomized and non-randomized studies, also suggesting a threshold indicating quality appraisal.

### 2.7. Risk of Bias Assessment

The risk of bias tool was assessed through the Revised Cochrane Risk of Bias 2.0 [36] and Revised Cochrane Risk of Bias 2.0 for crossover trials [37]. These scales, which are included in the Covidence tool, were used to assess the potential risk of bias in each study. Both tools [36,37] examine five potential sources of bias: (1) bias arising from the randomization process; (2) deviations from the intended intervention; (3) missing outcome data; (4) measurement of the outcome; and (5) selective outcome reporting. The latter also examines bias arising from period or carryover effects. Both tools generate an overall “risk rating” (i.e., “low risk”, “unclear risk”, “high risk”). 

## 3. Results

### 3.1. Literature Search and Study Selection

**Figure 1:**
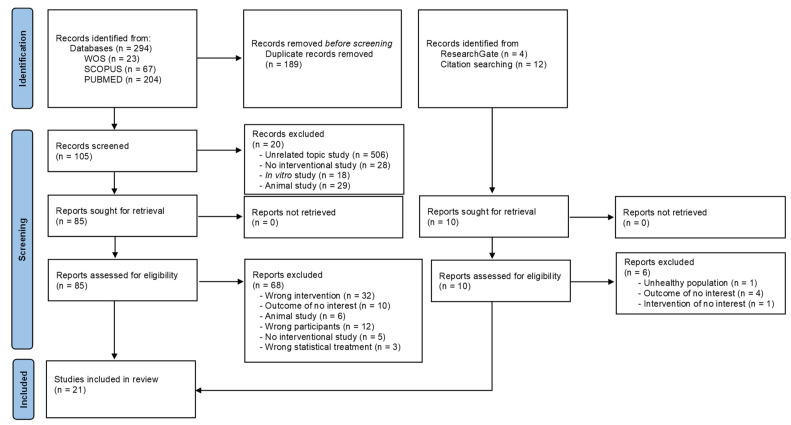
PRISMA flow diagram.

A flow diagram illustrating the article selection process is provided in Figure 1. Initially, a total of 294 results were obtained, which were trimmed to 105 results after eliminating duplicates (n = 189). Subsequently, a total of 105 titles and abstracts were screened. Of these, 85 full-text articles were originally identified as potentially relevant for this systematic review. However, after applying the eligibility criteria, 65 manuscripts did not fulfill these criteria. A total of 20 articles met all the inclusion criteria, but three were removed based on the statistical analysis used. On the other hand, four studies were obtained from other sources such as ResearchGate and reference lists of relevant studies. Hence, 21 articles were finally included in this systematic review [38,39,40,41,42,43,44,45,46,47,48,49,50,51,52,53,54,55,56,57,58].

### 3.2. Assessment of Methodological Quality

All included articles met the minimum requirements for methodological quality with a score equal to or greater than 13 corresponding to “very good” in McMaster (Table 2), and 8 points corresponding to “good” in the PEDro scale (Table 3). The main deficiencies of the included studies were evaluated in item 16 in McMaster and item 7 in the PEDro scale.

### 3.3. Risk of Bias Assessment

**Figure 2:**
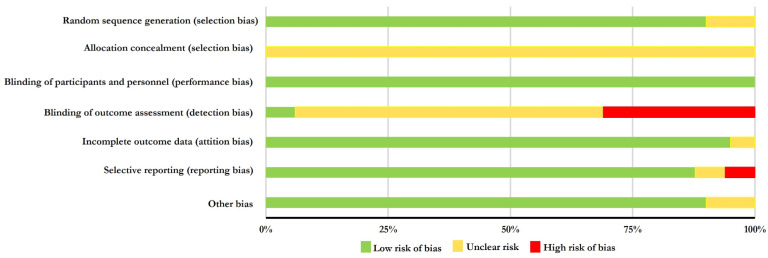
Most common problems found in the risk of bias of selected studies.

Table 4 shows the results of the risk of bias evaluation. None of the studies included in this systematic review were rated as overall “low risk” of bias. However, 13 studies [38,39,40,41,48,49,50,51,52,53,54,57,58] obtained “low risk” in five of the seven domains analyzed. Seven studies [42,43,52,53,56,57,58] were rated “high risk” of bias and 14 studies [38,39,40,41,44,45,46,47,48,49,50,51,54,55] had “unclear risk” noted. The most common problems were: (i) Allocation concealment (selection bias); (ii) Blinding of outcome assessment (detection bias) (Figure 2). Only one study justified the chosen sample size [44].

### 3.4. Characteristics of the Studies Included in the Systematic Review

As mentioned before, 21 human randomized clinical trials [38,39,40,41,42,43,44,45,46,47,48,49,50,51,52,53,54,55,56,57,58] were included in this systematic review focused on the analysis of melatonin supplementation in highly trained athletes (Table 5): nine studies with a parallel group design [42,44,47,52,53,55,56] and 14 with a crossover design [38,39,40,41,43,45,46,48,49,50,51,54,57,58]. Studies were performed on 354 participants: 62 women (17.5%) [42,43] and 292 men (82.5%) [38,39,40,41,42,44,45,46,47,48,49,50,51,52,53,54,55,56,57,58]. Among them, 247 individuals (69.8%) could be qualified as competitive or professional athletes [40,41,42,43,44,45,46,47,48,49,50,53,57], and 107 participants (31.2%) were categorized as highly trained athletes [38,39,51,52,54,55,56,58]. Thirteen studies [39,40,41,42,43,44,46,47,48,50,51,53,55,57] specified the sports modality: cycling [39], judo [40], volleyball [41], soccer [42,44,45,46,47,48,49,50,53], rowing [42], handball [43], and running [54,56] (Table 5). The 21 included studies [38,39,40,41,42,43,44,45,46,47,48,49,50,51,52,53,54,55,56,57,58] were carried out in eight countries: Brazil [38], USA [39], France [40,41], Poland [42], Finland [54], Turkey [51], Tunisia [43,44,45,46,47,48,49,50,57,58], and Spain [52,53,55,56]. 

### 3.5. Results Summary of the Studies Included in This Systematic Review Considering Health Circulating Biomarkers

Results of the studies included in this systematic review considering health biomarkers are shown in Table 5. These results are shown comparing the group supplemented with melatonin or intervention group (IG) versus the placebo group or control group (CG).

#### 3.5.1. Hematological Biomarkers

Melatonin supplementation does not modify the red blood cell (RBC) parameters [38,52,53,55,56], iron metabolism (transferrin, ferritin, serum iron) [56], plasma viscosity [55], white blood cell (WBC) count [38,52,53], immunoglobulin (Ig) M and G [53]. However, Cheickh et al. [41] reported significant decreases (*p* < 0.05) in WBC, particularly neutrophils and lymphocytes, after a 1-day acute supplementation with 10 mg of melatonin 2 h post-exercise. Furthermore, supplementation with 100 mg for 4 weeks significantly increased (*p* < 0.05) plasma IgA levels [53].

#### 3.5.2. Biochemical Parameters

*Blood Glucose*

Ten studies [42,43,44,45,49,52,53,54,56] evaluated blood glucose, but only two studies [42,43] obtained significant decreases (*p* < 0.05) in blood glucose through melatonin supplementation for 30 to 60 min pre-exercise compared to CG.

*Lipid Metabolism*

Parameters related to lipid profile were studied in seven articles [38,44,45,52,53,55,56]. Significant decreases (*p* < 0.05) in total cholesterol [38,52] and triglycerides [53], and a tendency to decrease (*p* > 0.05) in phospholipids [55] were reported in the melatonin group compared to the placebo group. Supplementation with melatonin before [44] or after [45,56] exercise did not modify HDL and/or LDL lipoprotein plasma levels versus CG.

*Kidney Function*

Kidney function was evaluated with four biomarkers such as uric acid [38,47,52,53,56], urea [38,44,45,52,53,56], creatinine [38,44,45,52,53,55,56], and total proteins [38,44,45,53,55]. Non-significant decreases (*p* > 0.05) were observed for uric acid [46], urea [44], and creatinine [44,52,55]. However, the study conducted by Farjallah et al. [45] described significant decreases (*p* < 0.05) in creatinine in the IG compared to the non-supplemented group.

*Liver Function*

Eight studies [41,44,45,46,52,53,55,58] included in this systematic review evaluated liver function. A significant (*p* < 0.05) decrease in aspartate aminotransferase (AST) [41,45], alanine aminotransferase (ALT) [45], and γ-glutamyl transferase (γ-GT) was observed in the IG, with the administration of melatonin in a single dose of 6 mg 30 min pre-exercise [45], or 10 mg 2 h after physical activity [41], compared with the CG. In addition, non-significant (*p* > 0.05) reductions in total bilirubin [46,55] and alkaline phosphatase [44,45] were also observed in the IG compared to placebo group. 

#### 3.5.3. Hormone Response

The administration of melatonin in highly trained athletes [54] or professional soccer players [53] did not induce changes in the pattern of cortisol [53,54], testosterone [53,54], and growth hormone [54] circulating levels.

#### 3.5.4. Inflammatory Response

The inflammatory response induced by high-intensity exercise was evaluated using five biomarkers in four of the clinical trials [41,42,55,58] included in this systematic review. The inflammatory biomarkers that reported significant decreases (*p* < 0.05) were C-reactive protein (CRP), IL-6 [42,55], and tumor necrosis factor-α (TNF-α) [55] when comparing both groups (CG vs. IG). Furthermore, in the anti-inflammatory parameters [55], a significant increase (*p* < 0.05) in the IL-1 receptor (IL-1Ra) and a tendency to increase (*p* > 0.05) in the soluble TNF-α receptor II (sTNF-α-RII) have been observed in the melatonin-supplemented group compared to the placebo group [55].

#### 3.5.5. Muscle Damage

Muscle damage was evidenced by the plasma concentration of the enzymes creatine kinase (CK) [38,41,44,46,47,52,53] and lactate dehydrogenase (LDH) [38,41,46,47,52,53,58]. Comparing IG vs. CG, significant decreases (*p* < 0.05) in both enzymes, CK [41,47,52] and LDH [41,47,52], were reported. Nevertheless, the two studies [44,46] conducted by Farjallah et al. reported a tendency to decrease (*p* > 0.05) in CK [44,46] and LDH [46] in the melatonin-supplemented group compared to CG in highly trained participants. 

#### 3.5.6. Antioxidant Status

*Antioxidant Enzymes*

The activity of the antioxidant enzyme superoxide dismutase (SOD) was significantly increased (*p* < 0.05) after 6 days of post-training supplementation with melatonin (5 mg) in professional soccer players [44,47]. In this line, catalase activity increased significantly (*p* < 0.05) after supplementation with a total dose of 15 mg of melatonin for 3 days before exercise in highly trained runners [55].

*Oxidative Stress Biomarkers*

All oxidative stress biomarkers evaluated, including malondialdehyde (MDA), oxidized low-density lipoprotein molecules (ox-LDLs), isoprostanes (8-iso-Prostaglandin F2α [8-iso-PGF2α or 5-F2t-IsoP]), homocysteine, advanced oxidation protein products (AOPP), lipid peroxidation, nitrites, 8-hydroxy-2′-deoxyguanosine (8-OHdG), decreased in the group supplemented with melatonin vs. the non-supplemented group, in eight clinical trials [41,42,44,45,46,52,53,55] included in this systematic review. Specifically, significant decreases (*p* < 0.05) in plasma levels were obtained in MDA [41,42,53], ox-LDLs [42], isoprostanes [45], homocysteine [41], AOPP [52], lipid peroxidation [52], and nitrites [52].

*Antioxidant Status*

Three antioxidant functionality biomarkers, including total antioxidant capacity (TAC) [56], oxygen radical absorption capacity (ORAC) [52], and total antioxidant status (TAS) [53,55] increased significantly (*p* < 0.05) in the IG compared to the CG. The dosage was carried out in a dose ranging between 6 mg [53] to 100 mg [52], in long administration periods of 4 weeks [52], short time of 3 days [55], and/or acute periods of 1 day [53,56], pre-exercise [53,55,56] or before rest/sleep [52] compared to CG.

*Glutathione Homeostasis*

Five studies evaluated glutathione (GSH) homeostasis [42,46,52,55,56]. Two studies (42,52] reported significant increases (*p* < 0.05) in GRd activity in IG compared to CG. GSH [42] and GPx activity [55,56] significant increased (*p* < 0.05] in the IG vs. CG. Two ratios, GSH/GSSG (oxidized GSH) and GPx/GRd, were evaluated by the study conducted by Leonardo-Mendonça et al. [52]. In both ratios, a significant decrease (*p* < 0.05) was observed after 4 weeks of melatonin supplementation (100 mg per day) compared to the placebo group in highly trained athletes [52].

#### 3.5.7. Perceptual and Cognitive Response

Regarding perceptual response, the rate of perceived exertion (RPE) decreased significantly (*p* < 0.05) in judo practitioners after the administration of 10 mg of melatonin 2 h before exercise compared to the non-supplemented group [40]. Also, RPE decreased non-significantly (*p* > 0.05) in professional soccer players after the single ingestion of 5 mg of melatonin 30 min before exercise [49]. Furthermore, the Visual Analogue Scale (VAS) to determine pain progression also decreased significantly (*p* < 0.05) in professional soccer players after 6 days of supplementation with 5 mg post-exercise with respect to the CG [47]. Two cognitive response assessment parameters were evaluated by Ghattassi et al. [48] with significant (*p* < 0.05) improvements in reaction time and vigilance test. 

#### 3.5.8. Physiological Parameters

Supplementation with 5 mg melatonin before exercise did not change physiological parameters such as rectal temperature [39], heart rate [49], diastolic blood pressure [49], and systolic blood pressure [49].

### 3.6. Summarized Results of the Studies Included in the Systematic Review Based on Sports Performance

Fifteen clinical trials [38,39,40,41,43,44,45,46,47,48,49,50,51,54,57] included in this systematic review analyzed sports performance parameters. Two studies [38,41] showed significant improvements (*p* < 0.05) in long-term continuous exercise. Aerobic capacity (Yo-Yo intermittent recovery test level 1 [YYIRT-1]) significantly increased (*p* < 0.05) in judo practitioners with a single dose of 10 mg of melatonin compared to CG [40]. Anaerobic capacity was assessed by blood lactate [43,45,49,51,54,57], anaerobic power [50,56], sprint performance [43,44,47], and agility tests [43,44,48,49,51]. In this sense, significant decreases (*p* < 0.05) in blood lactate [42,56], significant increases (*p* < 0.05) in anaerobic power [56], and a tendency to improve (*p* > 0.05) were described in agility [44] and sprint performance [44,47] in the IG compared to the placebo group.

A manual pressure strength or hand grip strength test, which evaluates the strength of the upper extremity [40,48,49,50,51], was performed in two studies obtaining significant (*p* < 0.05) [48] and modest (*p* > 0.05) [50] improvements in IG vs. CG with 5 mg of melatonin 30 min before exercise. However, with 8 mg of melatonin, with the same supplementation timing, manual pressure strength decreased significantly when compared to the placebo group [50].The strength and power of the lower extremities were evaluated by the ability to vertically jump [40], horizontally jump [40], squat jump [43,44,49,50], ball throw jump [43], countermovement jump [44,50,54], and the 5-jump test [40,44,48,50]. Only Cheikh et al. [40] have described significant (*p* < 0.05) improvements in jump performance in judo practitioners supplemented one day with 10 mg of melatonin 2 h post-exercise. In addition, medium power and peak power increased significantly (*p* < 0.05) in volleyball players after one day of supplementation with 10 mg of melatonin 2 h pre-exercise [41] vs. CG. However, with the single dose of 8 mg 30 min before exercise, jumping performance worsened significantly for squat jump and with a tendency for ball throw jump [50]. However, significant (*p* < 0.05) improvements were reported for some of these parameters in the IG [41] compared to the non-supplement group. 

### 3.7. Summarized Results of the Studies Included in the Systematic Review Based on Melatonin Parameters

#### 3.7.1. Melatonin Bioavailability

Plasma melatonin concentration increased significantly (*p* < 0.05) [53,54,55] and with a tendency to increase [42] (*p* > 0.05) in the IG compared to the CG.

#### 3.7.2. Adverse Effects

Only one study [42] evaluated adverse effects, observing that melatonin supplementation did not result in adverse reactions after administration

## 4. Discussion

This systematic review synthesizes information on the effects of melatonin supplementation on sports performance and circulating health biomarkers of highly trained athletes. To our knowledge, this study provides the first evidence-based review of 21 clinical trials [38,39,40,41,42,43,44,45,46,47,48,49,50,51,52,53,54,55,56,57,58] of melatonin supplementation to improve performance and health in competitive or professional athletes [40,41,42,43,44,45,46,47,48,49,50,53,57] and highly trained individuals [38,39,51,52,54,55,56,58]. Melatonin supplementation ranged between 5 mg [39,42,44,47,48,49,50] to 100 mg [52], administered acutely (single dose) [38,39,40,41,43,45,46,48,49,50,51,53,54,57,58], or continuously in periods ranging between 3 [55] to 30 days [42], pre-exercise [38,39,43,45,48,49,50,51,53,54,55,56,57,58] or post-exercise [40,41,42,44,46,47]. 

As a general description, the key improvements were reported in antioxidant status, inflammatory response, and reversing liver and muscle damage. Furthermore, melatonin supplementation had moderate effects on modulating glycemia, total cholesterol, triglycerides, and creatinine. In this context, it was difficult to determine the true effectiveness of melatonin to improve fitness as assessed by biomarkers of sports performance. Additionally, no melatonin-related adverse effects were reported. The variability in the duration and dose of supplementation and the type of exercise could contribute to the uncertainty in the results. For this reason, we have divided the systematic review into sections for a more correct research description. 

### 4.1. Melatonin Supplementation

The interventions carried out in the studies reviewed were performed by oral administration of exogenous melatonin in single doses made in laboratories (capsules [44,45,46,47,48,49,50,52,55,56,58] or tablets [38,39,40,41,43,54,57]). In 14 studies [38,40,41,43,44,45,46,47,48,49,50,54,55,56], melatonin was dispensed from a commercial trademark. The doses were heterogeneous, with 5 mg [39,42,44,47,48,49,50], 6 mg [38,43,45,46,53,54,57,58] and 10 mg [40,41,51] being the most used doses. Leonardo-Mendonça et al. [52] used the highest dose (100 mg melatonin) of all clinical trials included in this review. The 100 mg melatonin was the only dose above the recommended range in humans (3–20 mg) [59]. 

In this way, for the treatment of circadian rhythm sleep disorder, primary and/or secondary insomnia [60], doses between 3–10 mg/day [60] are used in healthy people, 3–20 mg/day for patients with cancer [61], and 2–10 mg/day for children [62]. In particular, 20–40 mg/day of melatonin were administered orally as a therapeutic adjuvant to patients with an advanced stage of metastatic cancer and no response to conventional treatments [63,64,65,66]. This indicates that the doses used in clinical interventions could justify the supplementation levels in highly trained athletes [38,39,40,41,42,43,44,45,46,47,48,49,50,51,52,53,54,55,56,57,58]. Nevertheless, doses for sport disciplines with very demanding exercise actions remain to be established [67], taking into account melatonin bioavailability [68]. In this context, the European Food Safety Authority (EFSA) has authorized doses of 1–2 mg/day [69].

The rapid intestinal absorption of exogenous melatonin allows it to reach its maximum plasma concentration after 40 min with a half-life ranging between 45 and 65 min after oral administration, while parenteral administration ranges from 0.5 to 6 min, with plasma concentrations lasting after many hours [60]. The pre-exercise times for oral supplementation ranged between 15 min [39] to 60 min [55], 30 min [38,43,45,48,49,50,53,57] being mostly used. This last time seems to be appropriate to obtain potential direct benefits on sports performance [70]. Taking melatonin after exercise could have some indirect effect on sports performance [70] due to the effects on athletes’ recovery [70] through antioxidant potential, ability to reduce muscle and liver damage, and anti-inflammatory actions [25,26,29,71]. These effects were significantly improved (*p* < 0.05) when comparing the non-supplemented groups in nine trials analyzed in this systematic review [41,42,44,45,47,52,53,55,56]. None of the studies included have determined plasma levels of endogenous melatonin. This indicates that future research would consider the diet analysis of athletes, since certain foods can significantly increase the concentration of melatonin, such as barley, rice, and tomatoes (in vegetables and cereals), cherries, strawberries, and grapes (in fruits), chicken or beef, olive oil, and nuts. Other compounds such as caffeine, vitamins, and/or minerals would modify the synthesis of melatonin [72,73].

No adverse events derived from melatonin supplementation were reported in the 21 studies [38,39,40,41,42,43,44,45,46,47,48,49,50,51,52,53,54,55,56,57,58] analyzed in this systematic review. However, athletes supplemented with 8 mg of melatonin showed significant (*p* < 0.05) harmful effects on sports performance in strength tests (hand grip test) and power tests (squat jump and countermovement jump tests) compared with the group supplemented with 5 mg of melatonin and the CG [50]. In this line, melatonin is a doping-free supplement according to the World Anti-Doping Agency [74].

### 4.2. Antioxidant Status

Exhaustive and extreme training leads to oxidative stress (OS), induced by the exacerbated production of RONS and the inability of the organism to maintain physiological homeostasis due to the breakdown of the redox balance [21]. OS causes cellular damage, especially to muscle, kidney, and liver tissues, triggering a hyperinflammatory response, resulting in decreased sports performance, overtraining, and premature fatigue [24]. Melatonin’s antioxidant properties prevent OS, avoiding tissular damage [28] and helping to reduce inflammation caused by RONS [27]. 

Furthermore, melatonin could improve the genetic expression of antioxidant enzymes, by modulating the nuclear factor erythroid 2-related factor 2 (Nrf2) pathway [68]. In highly trained athletes, SOD [44,47], CAT [55], and GPx [56,57] activities were significantly increased after supplementation with 5 mg [44,47] or 20 mg [56] after exercise or 15 mg before physical activity [55]. GSH is a key molecule of cellular homeostasis with a key role in the defense against OS [75]. Supplementation with 5 mg of melatonin for 30 days after exercise (30–60 min before going to sleep) significantly increased (*p* < 0.05) GSH levels compared to the placebo group [52]. A significant reduction (*p* < 0.05) in GRd [42,52], GSSG [52] and the GSSG/GSH ratio [52] was observed after melatonin supplementation compared to CG, which could show that highly trained endurance athletes have additionally increased GSH. These elevated levels of GSH could indicate an extra adaptation of antioxidant defenses for intense exercise in the presence of melatonin [76]. The significant increase (*p* < 0.05) [55,56] and tendency to increase (*p* > 0.05) [46] in GPx activity found here could reflect an antioxidant response against exercise-induced peroxides [77]. In this sense, melatonin significantly reduces (*p* < 0.05) advanced oxidation protein products (AOPP) and lipid peroxidation (LPO) levels [52].

In addition, as an indirect antioxidant action, melatonin negatively regulates pro-oxidative enzymes [29]. Melatonin suppresses inducible NO synthase (iNOS), responsible for nitric oxide (NO) synthesis, and lipoxygenase, responsible for hydroperoxidation of polyunsaturated fatty acids in cell membranes [29]. Leonardo-Mendonça et al. [52] have reported a significant decrease (*p* < 0.05) in LPO and nitrites in IG compared to CG. These results would show greater antioxidant protection at two levels [78]. The first level is against the rapid onset process of oxidation of membrane lipids and the second level is against the muscle damage process and the subsequent inflammatory response that induces the activation of iNOS and therefore the increase in NO [76]. Then, NO is capable of reacting with superoxide anions causing tissue damage [79]. In addition, the direct antioxidant action of melatonin protects the functions of many biological molecules such as DNA and proteins against OS. In previous reports, melatonin significantly reduced (*p* < 0.05) AOPP [52], LPO [52], and DNA damage [56]. AOPP, as an oxidation index of amino acids, is a key determinant in the inflammatory response [80]. 

Therefore, melatonin could be considered as a potent, versatile antioxidant, capable of preventing the overproduction of RONS induced by exercise, protecting the functions of molecules and tissue injuries from oxidative damage [29]. In the same line, antioxidant functionality, determined through TAC [56], TAS [53,55], and ORAC [52], increased significantly (*p* < 0.05) in the IG compared to the CG, before exercise with a dose range of between 6 mg [53] to 100 mg [52]. Melatonin has an antioxidant capacity higher than vitamin C and vitamin E [81,82], and similar to curcumin [20] or N-acetylcysteine [21].

### 4.3. Inflammatory Response 

Intense exercise induces physiological responses that disrupt homeostatic processes, leading to inadequate recovery and tissue repair [83]. Altogether, this gives rise to exercise-induced muscle damage (EIMD), which initiates with a local and systemic inflammatory hyper-response, inducing the release of inflammatory mediators [84]. Melatonin could be used as a sports supplement to control this inflammatory response and muscle damage [68], which would be beneficial for an athlete’s health status and sports performance. Regarding the biomarkers assessed (Table 5), melatonin improves inflammatory status in athletes through a significant reduction in the secretion of IL-6 [42,55] and TNF-α [55], as well as in acute phase reactants such as CRP [41,42] and a significant increase in anti-inflammatory mediators, including sTNF-α-RII and IL1-ra [55]. Melatonin modulates inflammation by inhibiting the activation of the NF-κB, JAK/STAT, and MAPK signaling pathways [85,86]. Interestingly, inhibition of the NF-κB pathway is the target of other sports supplements such as N-acetylcysteine [21], curcumin [20], probiotics [13], the antioxidants ascorbate (vitamin C), and α-tocopherol (vitamin E) [87], and *Tribulus terrestris* [88]. Thus, according to the results described in this systematic review, melatonin supplementation would modulate the inflammatory response in athletes through different protocols [41,42,55]. In this context, the dose used was 5 mg [42], 10 mg [41], and 15 mg [55], administered before [55] or after exercise [41,42] and with acute supplementation periods of 1 day [41] or longer periods of 3 days [55] or 30 days [42]. 

### 4.4. Tissue Damage

Regarding muscle damage, melatonin supplementation displayed significant reduction [41,47,52] or a tendency [44,46] to reduce the two biomarkers of muscle damage: CK and LDH. The modulating effect of damage in muscle tissue could be because of the antioxidant activity of melatonin, due to the attenuation of the peroxidation of lipids in cell membranes [29] or an increase in antioxidant enzymatic activity [68]. Furthermore, melatonin downregulates the expression of cyclooxygenase-2 (COX-2) by blocking the transcription of the COX-2 activator (p52) by inhibiting the activity of p300 histone acetyltransferase [89]. This activity could contribute to the reduction in CK and LDH plasma levels. Therefore, supplementation in a range of 5 mg to 100 mg after exercise in periods between 1 day and 28 days could attenuate SMID (skeletal muscle-induced damage) [41,47,52]. 

The hepatoprotective property of melatonin is due to the multimodal mechanism of inhibition of lipid peroxidation, increase in levels of antioxidant enzymes, and direct capacity to eliminate RONS [26,28]. Melatonin decreased all biomarkers of liver damage determined in the studies analyzed in this systematic review, protecting hepatocytes against OS. Furthermore, significant reduction in AST [41,45], ALT [45], and γ-GT [45], or tendency to decrease of γ-GT [44], total bilirubin [46,55], and alkaline phosphatase [44,45], may corroborate the non-toxicity of melatonin supplementation. Single dose supplementation of 6 mg [45] or 10 mg [41] after exercise was shown to be capable of playing a hepatoprotective role against intense exercise in highly trained athletes, with no side effects.

Kidney biomarkers increase after intense exercise [83]. The cytoprotective property of melatonin on the renal system is manifested with a significant decrease in creatinine (6 mg single-dose after exercise) [45], and a tendency to decrease in uric acid [46], urea [44], and creatinine [44,52,55] in the supplemented athletes compared to the placebo group. Therefore, the administration of melatonin seems to be safe, improving liver and kidney functions [89]. The safety in the administration of melatonin is consistent with the results reported in the analyzed studies (Table 5) as it does not modify main circulating parameters and biomarkers [38,52,53,55,56]. 

### 4.5. Immune System

Intense exercise has a temporary negative impact on immune function, inducing a marked leukocytosis, and a moderate lymphocytosis, increasing post-exercise and recovering after 1–3 h [83]. The immunomodulatory action of melatonin is established through the melatonin 1 (MT1) receptors, inhibiting NF-κB. This results in reduced expression of proinflammatory enzymes such as iNOS and the cytokines as IL-2, TNF-α, interferon-γ (IFN-γ), and Granulocyte-Macrophage Colony Stimulating Factor. Furthermore, melatonin through MT1 would increase the levels of TGF-β (Transforming Growth Factor-β) together with IL-4, modulating the activity of immune cells [90,91,92]. 

Transitory immunosuppression that occurs after strenuous exercise results in a transient decrease in WBC function. This situation favors infections by viruses and bacteria particularly in the upper respiratory tract [93]. In this context, upper respiratory tract infections (URTIs) are the most common disturbances in competitors of aerobic disciplines that require greater speed and depth in breathing [94]. In this context, melatonin could be a key defense supplement [95].

### 4.6. Hormonal Response

It has been shown that prolonged and intensive exercise induces a dysfunction of the hormonal pattern by decreasing the secretion of testosterone and increasing the secretion of cortisol. These alterations in hormonal profile generate a situation of slight catabolism that negatively influences recovery capacity and decreases sports performance [10]. Melatonin did not modify the levels of testosterone [53,54], cortisol [53,54], or total circulating proteins. In addition, melatonin did not modify the concentration of growth hormone [54]. It appears that melatonin supplementation has no ability to modulate hormone actions.

### 4.7. Physical Performance 

The physiological-metabolic pathways of melatonin that involve antioxidant, anti-inflammatory, immunomodulatory, and tissue damage-attenuating actions described in this systematic review could make it a sports nutritional supplement of interest for highly trained athletes to increase performance. Also, melatonin supplementation increases glucose in the muscle, improves lipid profile (evidenced by significant decrease in total cholesterol [38,52] and triglycerides [53]), and reduces body mass, contributing to better adaptation to demanding efforts [70]. However, no direct relationship between melatonin supplementation and sports performance has been described [31,70]. In this way, melatonin produced a decrease in strength and power [31]. This could be due to the depressive effects of this hormone on the central nervous system or to hypoglycemia reported in other studies [42,43]. Six studies included in this review have described significant benefits of melatonin supplementation compared to CG in tests of aerobic capacity [38,41], anaerobic capacity [42,56], balance [56], lower extremity strength/power [40], fatigue index [41], RPE [40], VAS [46], and time of reaction [47]. The divergences in sports performance parameters could be due to the type and duration of supplementation, or the type and duration of exercise, which could condition the actions of melatonin on performance tests.

### 4.8. Limitations and Strengths

Limitations of the systematic review include: (i) A restricted number of manuscripts met the inclusion criteria. (ii) The high heterogeneity of the studies: the outcomes, the dosage of melatonin supplementation, and the type of physical activity performed by participants. This heterogeneity prevented a complete meta-analysis study and implies caution when interpreting the results. (iii) The sample studied is exclusively of highly trained athletes, so generalization to other populations may only be made with caution. In this line, it would be necessary to increase the number of studies including a larger number of male and female subjects, including physically active people, to complete the observations carried out with trained athletes. Nevertheless, the evidence obtained from studies in highly trained athletes strongly supports the health properties of melatonin. 

On the other hand, these limitations are not the consequence of an incomplete revision system. This systematic approach followed the PRISMA rules; the search was run using three databases, Medline (PubMed), SCOPUS and WOS, and included grey literature. At the same time, we used the modified McMaster methodological quality assessment tool [35] and PEDro scale [34]. The risk of bias was tested by using the Revised Cochrane Risk of Bias 2.0 [36] and Revised Cochrane Risk of Bias 2.0 for crossover trials [37]. All these tools ensure that all selected records met minimum quality criteria and included health biomarkers and sports performance parameters that are commonly used in research studies in the field of sports nutrition.

## 5. Practical Applications

This research could be of interest to sports doctors, dieticians, and coaches since it represents an improvement in the supplementation strategies necessary for highly trained athletes. In general, melatonin supplementation could be used during periods of high demand, tournaments, or competitive events to accelerate recovery of muscle function and counteract oxidative state, inflammatory response, and reverse liver and muscle damage. Nutrition is the basis of performance and health for athletes, especially in strenuous sports disciplines that use all energy systems in practice and competition. Therefore, it seems reasonable to implement nutritional aids that allow athletes to cover the extra nutritional requirements, optimizing their health and sports performance, which are also consistent with the principles of rational nutrition [96]. Regarding melatonin, an efficient dose of melatonin cannot be achieved only with food; although there is an indirect effect of diet, supplementation is necessary in the context of “food first”. To do this, it is necessary to take an inclusive nutrition and lifestyle approach to optimize plasma melatonin concentration. To ensure healthy levels of melatonin in highly-trained athletes, larger interventions could be implemented, including lifestyle modifications with appropriate exposure to light, selection of dietary patterns and specific foods, and, finally, supplementation only when necessary [97].

## 6. Conclusions

The results presented in the studies in this systematic review reported that melatonin has a high safety profile. Regarding the improvements in certain health biomarkers, the pleiotropic effect of melatonin may act to counteract and mitigate some of the effects induced by high-intensity physical exercise performed by high-level athletes such as OS, inflammation, and SMID. However, the molecular and physiological mechanisms of melatonin to directly improve sports performance remains to be determined. Melatonin supplementation could act indirectly to improve performance by preventing tissue damage and helping to reduce inflammation caused by RONS, restoring circulating biomarkers that go out of the normal range in highly trained athletes when performing very demanding exercises.

## Figures and Tables

**Table 1:** PICOS criteria for inclusion and exclusion studies.

Criteria	Inclusion Criteria	Exclusion Criteria
Population “P”	The sample must be composed of men and/or women trained athletes.	Participants receiving any type of medication or treatment; unhealthy individuals with gastrointestinal problems, with inflammatory and/or immunological pathologies, with musculoskeletal pathology. In general, individuals with chronic diseases.
	Individuals within the sample must be explicitly referred to as “trained athletes” in the manuscript or meet the criteria to be classified as “physically active” for exceeding the exercise recommendations of the American College of Sports Medicine (ACSM).	
Intervention “I”	Include a supplementation intervention that should involve the use of melatonin in monotherapy, with clear information on dosage and duration of melatonin supplementation.	Administration together with other nutritional supplements.
Comparison “C”	Include either a placebo or control group (parallel group studies design) or experimental condition (cross-over studies design).	With other doses of melatonin, or with other nutritional supplements.
Outcomes “O”	Any parameter related to sports performance, and/or biomarkers related to health status, that reports on the bioavailability and adverse effects of melatonin.	None.
Study design “S”	Human intervention studies, randomized controlled trials, and/or randomized controlled crossover trials.	Observational studies and studies that used a targeted analytical approach.

**Table 2:** Results of the methodological quality assessment of included studies—McMaster Critical Review Form for Quantitative Studies [35].

Study, Year	Item																Total	%	Quality Score
	1	2	3	4	5	6	7	8	9	10	11	12	13	14	15	16			
Beck et al., 2018 [38]	1	1	1	1	1	1	1	1	1	1	0	1	1	1	1	0	14	87.5	VG
Brandeberger et al., 2018 [39]	1	1	1	1	1	1	1	1	1	1	0	1	1	1	1	0	14	87.5	VG
Cheikh et al., 2018 [40]	1	1	1	1	1	1	1	1	1	1	1	1	1	1	1	0	15	93.8	E
Cheikh et al., 2020 [41]	1	1	1	1	1	1	1	1	1	1	1	1	1	1	1	0	15	93.8	E
Czucejko et al., 2019 [42]	1	1	1	1	1	1	0	1	1	1	1	1	1	1	1	0	14	87.5	VG
Farjallah et al., 2018 [43]	1	1	1	0	1	1	1	1	1	1	1	1	1	1	1	0	14	87.5	VG
Farjallah et al., 2022 [44]	1	1	1	1	1	1	1	1	1	1	1	1	1	1	1	1	16	100	E
Farjallah et al., 2023 [45]	1	1	1	1	1	1	1	1	1	1	1	1	1	1	1	0	15	93.8	E
Farjallah et al., 2022 [46]	1	1	1	1	1	1	1	1	1	1	1	1	1	1	1	1	16	100	E
Farjallah et al., 2020 [47]	1	1	1	0	1	1	1	1	1	1	1	1	1	1	1	0	14	87.5	VG
Ghatassi et al., 2016 [48]	1	1	1	1	1	1	1	1	1	1	1	1	1	0	1	1	15	93.8	E
Ghatassi et al., 2024 [49]	1	1	1	1	1	1	1	1	1	1	1	1	1	0	1	1	15	93.8	E
Ghatassi et al., 2014 [50]	1	1	1	0	1	1	1	1	1	1	1	1	1	1	0	0	13	81.3	VG
Khaleghi-Mamaghani et al., 2021 [51]	1	1	1	0	1	1	1	1	1	1	1	1	1	1	0	0	13	81.3	VG
Leonardo-Mendonça et al., 2017 [52]	1	1	1	1	1	1	1	1	1	1	0	1	1	1	1	0	14	87.5	VG
Maldonado et al., 2012 [53]	1	1	1	1	1	1	1	1	1	1	0	1	1	1	1	0	14	87.5	VG
Mero et al., 2006 [54]	1	1	1	0	1	1	1	1	1	1	1	1	1	1	0	0	13	81.3	VG
Ochoa et al., 2011 [55]	1	1	1	0	1	1	1	1	1	1	1	1	1	1	0	0	13	81.3	VG
Ortiz-Franco et al. 2017, [56]	1	1	1	1	1	1	1	1	1	1	0	1	1	1	1	0	14	87.5	VG
Paryab et al., 2021 [57]	1	1	1	1	1	1	1	1	1	1	1	1	1	1	1	0	15	93.8	VG
Souissi et al., 2018 [58]	1	1	1	0	1	1	1	1	1	1	1	1	1	1	1	0	14	87.5	VG

Abbreviations: 0 = unfulfilled criterion; 1 = fulfilled criterion; E = excellent; VG = very good. McMaster Critical Review Items: Item 1: study purpose; item 2: literature review; item 3: study design; item 4: blinding; item 5: sample description; item 6: sample size; item 7: ethics and consent; item 8: validity of outcomes; item 9: reliability of outcomes; item 10: intervention description; item 11: statistical significance; item 12: statistical analysis; item 13: clinical importance; item 14: conclusions; item 15: clinical implications; item 16: study limitations.

**Table 3:** Results of the methodological quality assessment of included studies—PEDro scale [34].

Study, Year	1	2	3	4	5	6	7	8	9	10	11	Total	%	Quality Score
Beck et al., 2018 [38]	1	1	1	1	1	0	0	1	1	1	1	9	81.8	G
Brandeberger et al., 2018 [39]	1	1	1	1	1	1	0	1	1	0	0	8	72.7	G
Cheikh et al., 2018 [40]	1	1	1	1	1	1	0	1	1	1	1	10	90.9	E
Cheikh et al., 2020 [41]	1	1	1	1	1	1	0	1	1	1	1	10	90.9	E
Czucejko et al., 2019 [42]	1	1	1	1	1	1	0	1	1	1	1	10	90.9	E
Farjallah et al., 2018 [43]	1	1	1	1	1	0	0	1	1	1	1	9	81.8	G
Farjallah et al., 2022 [44]	1	1	1	1	1	1	0	1	1	1	1	10	90.9	E
Farjallah et al., 2023 [45]	1	1	1	1	1	1	0	1	1	1	1	10	90.9	E
Farjallah et al., 2022 [46]	1	1	1	1	1	1	1	1	1	1	1	11	100	E
Farjallah et al., 2020 [47]	1	1	1	1	1	0	0	1	1	1	1	9	81.8	G
Ghatassi et al., 2016 [48]	1	1	1	1	1	1	0	1	1	1	1	10	90.9	E
Ghatassi et al., 2024 [49]	1	1	1	1	1	1	0	1	1	1	1	10	90.9	E
Ghatassi et al., 2014 [50]	1	1	1	1	1	0	0	1	1	1	1	9	81.8	G
Khaleghi-Mamaghani et al., 2021 [51]	1	1	1	1	1	0	0	1	1	1	1	9	81.8	G
Leonardo-Mendonça et al., 2017 [52]	1	1	1	1	1	0	0	1	1	1	1	9	81.8	G
Maldonado et al., 2012 [53]	1	1	1	1	1	0	0	1	1	1	1	9	81.8	G
Mero et al., 2006 [54]	1	1	1	1	1	0	0	1	1	1	1	9	81.8	G
Ochoa et al., 2011 [55]	1	1	1	1	1	1	0	1	1	1	1	10	90.9	E
Ortiz-Franco et al., 2017 [56]	1	1	1	1	1	1	0	1	1	1	1	10	90.9	E
Paryab et al., 2021 [57]	1	1	1	1	1	1	0	1	1	1	1	10	90.9	E
Souissi et al., 2018 [58]	1	1	1	1	1	0	0	1	1	1	1	9	81.8	G

Abbreviations: 0 = unfulfilled criterion; 1 = fulfilled criterion; E = excellent; G = good. PEDro questionnaire items = 1: eligibility criteria; 2: random assignment; 3: hidden allocation; 4: baseline comparison; 5: blind subjects; 6: blind therapist; 7: blind evaluators; 8: adequate follow-up; 9: intention-to-treat analysis; 10: comparison between groups; 11: point estimates and variability.

**Table 4:** Risk of bias of selected studies.

Study	Random Sequence Generation (Selection Bias)	Allocation Concealment (Selection Bias)	Blinding of Participants and Personnel (Performance Bias)	Blinding of Outcome Assessment (Detection Bias)	Incomplete Outcome Data (Attrition Bias)	Selective Reporting (Reporting Bias)	Other Bias	Overall Risk Rating	Sample Size Was Justified
Beck et al., 2018 [38]	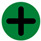	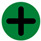	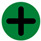	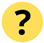	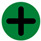	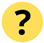	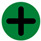	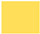	N
Brandeberger et al., 2018 [39]	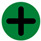	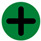	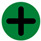	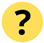	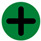	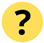	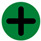	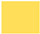	N
Cheikh et al., 2018 [40]	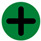	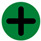	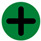	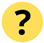	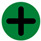	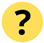	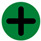	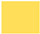	N
Cheikh et al., 2020 [41]	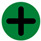	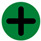	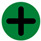	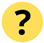	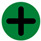	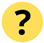	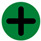	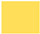	N
Czucejko et al., 2019 [42]	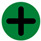	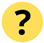	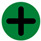	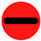	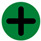	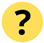	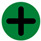	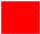	N
Farjallah et al., 2018 [43]	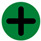	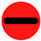	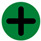	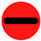	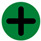	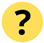	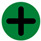	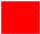	N
Farjallah et al., 2022 [44]	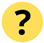	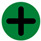	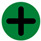	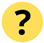	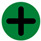	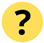	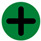	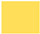	Y
Farjallah et al., 2023 [45]	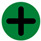	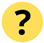	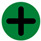	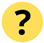	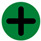	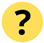	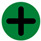	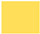	N
Farjallah et al., 2022 [46]	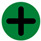	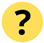	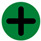	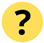	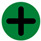	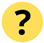	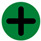	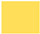	N
Farjallah et al., 2020 [47]	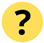	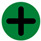	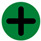	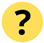	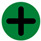	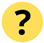	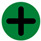	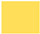	N
Ghatassi et al., 2016 [48]	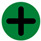	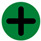	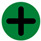	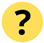	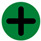	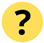	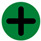	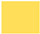	N
Ghatassi et al., 2024 [49]	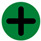	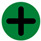	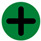	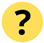	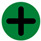	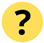	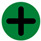	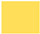	N
Ghatassi et al., 2014 [50]	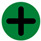	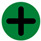	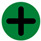	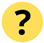	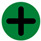	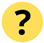	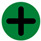	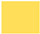	N
Khaleghi-Mamaghani et al., 2021 [51]	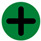	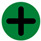	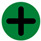	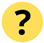	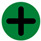	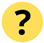	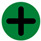	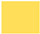	N
Leonardo-Mendonça et al., 2017 [52]	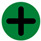	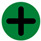	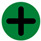	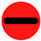	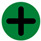	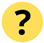	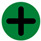	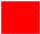	N
Maldonado et al., 2012 [53]	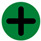	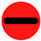	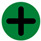	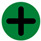	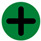	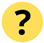	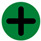	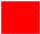	N
Mero et al., 2006, Finland [54]	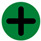	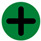	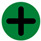	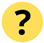	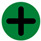	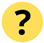	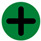	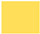	N
Ochoa et al., 2011 [55]	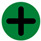	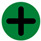	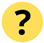	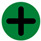	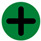	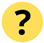	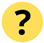	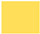	N
Ortiz-Franco et al., 2017 [56]	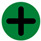	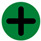	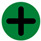	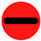	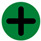	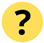	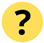	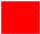	N
Paryab et al., 2021 [57]	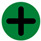	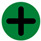	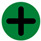	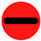	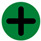	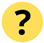	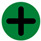	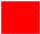	N
Souissi et al., 2018 [58]	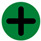	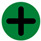	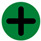	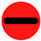	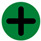	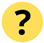	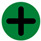	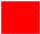	N

Risk of bias as assessed using the Revised Cochrane Risk of Bias tool (RoB 2.0) [36] and the RoB 2.0 for crossover trials [37]. Green = low risk of bias; Yellow = unclear risk of bias; Red = high risk of bias; N = No; Y = Yes.

**Table 5:** Studies included in the systematic review of the effect of melatonin supplementation on circulating health biomarkers and sports performance tests.

First Author, Year of Publication and Country (Reference)	Study Design	Characteristics of Participants: Baseline Sample (n, Sex, and Sport Discipline), Age (Mean ± SD), Anthropometric Parameters (Height, Weight, BMI, Body Fat and Muscle Mass) (Mean ± SD), Maximal Oxygen Consumption/Maximal Aerobic Speed (Mean ± SD) and Withdrawals	Intervention	Outcomes	Results IG vs. CG
Beck et al., 2018, Brazil [38]	Randomized, double-blind, placebo-controlled crossover trial	11 ♂ trained athletes in multiple sports (soccer, handball, basketball, cycling) Age: 24.18 ± 3.92 years Height: 182 ± 5 cm Weight: 87.07 ± 12.48 kg BMI: 26.18 ± 3.63 kg/m^2^ Body Fat: 16.28 ± 5.77% Study withdrawals: 0	Melatonin: 6 mg tablets (2 mg calcium, 55 mg phosphorus) (Optimum Nutrition, Inc., Downers Grove, IL, USA) Placebo: same conditions with no melatonin 30 min before exercise 1 day 06:00–09.00 p.m. Washout period: 48 to 72 h	RCB Hemoglobin Hematocrit WBC Neutrophils Lymphocytes Monocytes Uric Acid Urea Creatinine Total Proteins Glucose Total cholesterol Triglycerides CK LDH Time to exhaustion test	↔ RCB ↔ Hemoglobin ↔ Hematocrit ↔ WBC ↔ Neutrophils ↔ Lymphocytes ↔ Monocytes ↔ Uric Acid ↔Urea ↔ Creatinine ↔ Total Proteins ↔ Glucose ↓ * Total cholesterol ↔ Triglycerides ↔ CK ↔ LDH ↑ * Time to exhaustion test
Brandeberger et al., 2018, USA [39]	Randomized, double-blind, placebo-controlled, crossover trial	10 ♂ endurance-trained cyclists Age: 25.1 ± 4.0 years Height: 176 ± 7.1 cm Body Fat: 9.2 ± 13.2% VO_2_max: 62.7 ± 6.3 mL/kg/min Study withdrawals: 0	Melatonin: 5 mg Tablets (NR) Placebo: (multivitamin) similar size and shape to the melatonin 15 min before exercise 1 day 02:00–06.00 p.m. Washout period: ≥7 days	Rectal temperature RPE Medium Power Average Cycling Cadence 32.2 km cycling time trial	↔ Rectal temperature ↔ RPE ↔ Medium Power ↔ Average Cycling Cadence ↔ 32.2 km cycling time trial
Cheikh et al., 2018, France [40]	Randomized, double-blind, placebo-controlled crossover trial	10 ♂ national-level judo competitors (currently training ~8 h/week) Age: 15.4 ± 0.3 years Weight: 60.6 ± 5.7 kg Height: 167.9 ± 6.9 cm BMI: 21.21 ± 2.5 kg/m^2^ Study withdrawals: 0	Melatonin: 10 mg Tablets MEL-10 mg (Jamieson lab Toronto, Montreal, Vancouver, Canada N8W5B5) Placebo: starch and cellulose 2 h after exercise (15 min before bedtime) 1 day 10:00 p.m. Washout period: 7 days	RPE Hooper index (Fatigue) YYIRT-1 Manual pressure force 5-Jump Test Vertical jump Horizontal jump	↓ * RPE ↓ Hooper Index ↑ * YYIRT-1 ↔ Manual pressure force ↑ 5-Jump Test ↔ Vertical jump ↔ Horizontal jump
Cheikh et al., 2020, France [41]	Randomized, double-blind, placebo-controlled crossover trial	14 ♂ volleyball players from Tunisian league 1 (training 4 days per week for an average of 1.5–2 h) Age: 14.5 ± 0.52 years Weight: 65.68 ± 7.72 kg Height: 181.57 ± 7.38 cm BMI: 21.21 ± 2.5 kg/m^2^ Study withdrawals: 0	Melatonin: 10 mg Tablets MEL-10 mg (Jamieson lab Toronto, Montreal, Vancouver, Canada N8W5B5) Placebo: starch and cellulose 2 h after exercise (15 min before bedtime) 1 day 10:00 p.m. Washout period: 7 days	WBC Neutrophils Lymphocytes CK LDH AST CRP MDA Homocysteine Fatigue Inex Medium Power Peak Power Time trial	↓ * WBC ↓ * Neutrophils ↓ * Lymphocytes ↓ * CK ↓ * LDH ↓ * AST ↓ * CRP ↓ * MDA ↓ *Homocysteine ↓ * Fatigue Inex ↑ * Medium Power ↑ * Peak Power ↓ * Time trial
Czucejko et al., 2019, Poland [42]	Randomized, double-blind, placebo-controlled, parallel group trial	81 ♂ and ♀ IG: 47 ♀ second and third league soccer players (Zawisza Bydgoszcz Sports Club, Bydgoszcz, Poland), 19 ♂ rowers (Bydgoszcz Rowing Club, Poland); CG 15 ♂ non-training young adults Melatonin Group: Age: 20.95 ± 2.5 years Weight: 89.7 ± 8.5 kg Height: 1.85 ± 0.2 m BMI: 26.2 ± 0.2 kg/m^2^ Control Group: Age: 20.50 ± 2.0 years Weight: 82.1 ± 6.5 kg Height: 1.82 ± 0.11 m BMI: 24.8 ± 0.1 kg/m^2^ Study withdrawals: 0 66 participants in IG 15 participants in CG	Melatonin: Orally in a single dose 5 mg melatonin (NR) daily Placebo: NR 1 h before bedtime 30 days No adverse effects were reported	Glucose IL-6 CRP MDA 8-iso-PGF2α Isoprostane Ox-LDLs SOD GRd GSH GPx Melatonin	↓ * Glucose ↓ * IL-6 ↓ * CRP ↓ * MDA ↓ * 8-iso-PGF2α Isoprostane ↓ * Ox-LDLs ↔ SOD ↓ * GRd ↑ * GSH ↔ GPx ↑ Melatonin
Farjallah et al., 2018, Tunisia [43]	Randomized, double-blind, placebo-controlled crossover trial	15 ♀ elite athletes (Tunisian handball national team) Age: 17.4 ± 0.4 years Weight: 76.4 ± 5.6 kg Height: 176.0 ± 4.2 cm Study withdrawals: 0	Melatonin: 6 mg Tablets quick release (Jamieson Laboratories Toronto, Montreal, Canada) Placebo: lactose, starch, and cellulose 30 min before exercise 4:00 and 4:30 p.m. 1 day Washout period: 2 weeks	Glucose RPE Blood Lactate Modified agility T-test Squat jump Counter movement jump Maximum standing ball throw velocity test Maximum jump ball throw velocity test 20-m sprint	↓ * Glucose ↔ RPE ↓ * Blood Lactate ↔ Modified agility T-test ↔ Squat jump ↔ Counter movement jump ↔ Maximum standing ball throw velocity test ↔ Maximum jump ball throw velocity test ↔ 20-m sprint
Farjallah et al., 2022, Tunisia [44]	Randomized, double-blind, placebo-controlled, parallel group trial	24 ♂ professional soccer players from Tunisian first league Age: 18.8 ± 1.3 years Weight: 70.0 ± 10.6 kg Height: 181 ± 8 cm BMI: 21.27 ± 1.87 kg/m^2^ Study withdrawals: 4 10 participants in IG 10 participants in CG	Melatonin: 5 mg Capsules (Jamieson Laboratories Toronto, Montreal, Canada) Placebo: lactose, starch, and cellulose After exercise 6 days 7.00 p.m.	Creatinine Urea Glucose Total Cholesterol HDL LDL Triglycerides Total Proteins γ-glutamyl transferase Alkaline Phosphatase CK AOPP SOD Squat jump Countermovement jump 5-jump test Modified agility T-test 20-m sprint	↓ Creatinine ↓ Urea ↔ Glucose ↔ Total Cholesterol ↔ HDL ↔ LDL ↔ Triglycerides ↔ Total Proteins ↓ γ-glutamyl transferase ↓ Alkaline Phosphatase ↓ CK ↓ AOPP ↑ * SOD ↑ Squat jump ↑ Countermovement jump ↑ 5-jump test ↓ Modified agility T-test ↓ 20-m sprint
Farjallah et al., 2023, Tunisia [45]	Randomized, double-blind, placebo-controlled, parallel group trial	12 ♂ professional soccer players from Tunisian first league (soccer experience from 5–7 years) Age: 17.54 ± 0.78 years, Weight: 70.31 ± 3.86 kg Height: 1.80 ± 0.08 m Maximal Aerobic Speed: 16.85 ± 0.63 km/h Study withdrawals: 0	Melatonin: 6 mg Tablets quick release (Jamieson Laboratories Toronto, Montreal, Canada) Placebo: lactose, starch, and cellulose 30 min before exercise 5:00 ± 0:30 p.m. 1 day Washout period: 48 h	WBC Neutrophils Lymphocytes Monocytes Glucose Total Cholesterol HDL LDL Triglycerides Creatinine Urea Total Proteins AST ALT γ-glutamyl transferase Alkaline Phosphatase Blood Lactate Heart Rate Time to exhaustion Distance covered	↔ WBC ↔ Neutrophils ↔ Lymphocytes ↔ Monocytes ↔ Glucose ↔ Total Cholesterol ↔ HDL ↔ LDL ↔ Triglycerides ↓ * Creatinine ↔ Urea ↔ Total Proteins ↓ * AST ↓ * ALT ↓ * γ-glutamyl transferase ↓ Alkaline Phosphatase ↔ Blood Lactate ↓ Heart Rate ↑ Time to exhaustion ↑ Distance covered
Farjallah et al., 2022, Tunisia [46]	Randomized, double-blind, placebo-controlled crossover trial	13 ♂ professional soccer players from Tunisian first league Age: 17.5 ± 0.8 years Weight: 70.0 ± 3.9 kg Height: 180 ± 8 cm Maximal Aerobic Speed: 16.85 ± 0.63 km/h Study withdrawals: 0	Melatonin: 6 mg Capsules (Jamieson Laboratories Toronto, Montreal, Canada) Placebo: lactose, starch, and cellulose 10 min after exercise 1 day 05.00 p.m.–00.30 a.m. Washout period: 2 days	Uric Acid Total Bilirubin CK LDH MDA AOPP SOD GPx GRd RPE Running exercise test	↓ Uric Acid ↓ Total Bilirubin ↓ CK ↓ LDH ↓ MDA ↓ AOPP ↑ SOD ↑ GPx ↔ GRd ↔ RPE ↔ Running exercise test
Farjallah et al., 2020, Tunisia [47]	Randomized, double-blind, placebo-controlled, parallel group trial	20 ♂ professional soccer players from Tunisian first league Age: 18.9 ±1.3 years Weight: 70.1 ± 10.6 kg Height: 180 ± 1.0 cm Study withdrawals: 0 10 participants in IG 10 participants in CG	Melatonin: 5 mg Capsules (Jamieson Laboratories Toronto, Montreal, Canada) Placebo: lactose, starch, and cellulose After exercise 6 days 7.00 p.m.	CK LDH MAD SOD VAS Repeated sprint ability test	↓ * CK ↓ * LDH ↓ * MAD ↑ * SOD ↓ * VAS ↓ Repeated sprint ability test
Ghatassi et al., 2016, Tunisia [48]	Randomized, double-blind, placebo-controlled crossover trial	12 ♂ soccer players (Tunisian League 3) Age: 17.9 ± 1.3 years Weight: 62.0 ± 8.8 kg Height: 174 ± 6 cm	Melatonin: 5 mg Capsules (Jamieson Laboratories Toronto, Montreal, Canada) Placebo: lactose, starch, and cellulose 30 min before exercise 1 day 07.30 a.m. Washout period: 36 h	Medicine-ball throw Manual pressure force 5-jump test Agility T-test Reaction time Vigilance tests	↑ * Medicine-ball throw ↑ * Manual pressure force ↔ 5-jump test ↔ Agility T-test ↓ * Reaction time ↓ * Vigilance tests
Ghatassi et al., 2024, Tunisia [49]	Randomized, double-blind, placebo-controlled crossover trial	12 ♂ soccer players (Tunisian League) Age: 22.9 ± 1.3 years Weight: 72.0 ± 8.8 kg Height: 1.80 ± 0.05 m	Melatonin: 5 mg Capsules (Jamieson Laboratories Toronto, Montreal, Canada) Placebo: lactose, starch, and cellulose 30 min before exercise 1 day 07.30 a.m. Washout period: 48 h	Glucose Hooper’s index Manual pressure force Squat jump RPE Reaction time Vigilance Test Blood lactate Peak Power Average Power Modified agility T-test	↔ Glucose ↓ Hooper’s index ↑ Manual pressure force ↑ Squat jump ↓ RPE ↓ Reaction time ↔ Vigilance Test ↔ Blood lactate ↑ Peak Power ↑ Average Power ↔ Modified agility T-test
Ghatassi et al., 2014, Tunisia [50]	Randomized, double-blind, placebo-controlled crossover trial	12 ♂ soccer players (Tunisian League 3) Age: 17.9 ± 1.3 years Weight: 62.0 ± 8.8 kg Height: 174 ± 6 cm	Melatonin: 5 mg or 8 mg Capsules (Jamieson Laboratories Toronto, Montreal, Canada) Placebo: lactose, starch, and cellulose 30 min before exercise 07.30 a.m. 1 day Washout period: NR 3 test sessions at 9:00 p.m. on different days	Medicine-ball throw Manual pressure force 5-jump test Squat jump Counter movement jump Agility T-test Reaction time Vigilance tests	*Melatonin: 5 mg* ↔ Medicine-ball throw ↑ Manual pressure force ↔ 5-jump test ↔ Squat jump ↔ Counter movement jump ↔ Agility T-test *Melatonin: 8 mg* ↓ Medicine-ball throw ↓ * Manual pressure force ↓ 5-jump test ↓ * Squat jump ↓ * Counter movement jump ↔ Modified agility T-test
Khaleghi-Mamaghani et al., 2021, Turkey [51]	Randomized, double-blind, placebo-controlled crossover trial	10 ♂ highly trained (3–4 days per week for an average of 2-h training in a day) Age: 23.4 ± 1.71 years Weight: 74.28 ± 6.69 kg Height: 176 ± 6.42 cm BMI: 23.96 ± 1.63 kg/m^2^ Body Fat: 13.4 ± 2.75%	Melatonin: 10 mg (NR) Placebo: (NR) 30 to 45 min after exercise 1 day 11.00 a.m. Washout period: 1 week	Heart Rate DBP SBP Blood Lactate Reaction time Dynamic balance Static balance Jump strength Manual pressure force Squat Bench press Anaerobic peak power Anaerobic minimum power Average power Fatigue index	↓ Heart Rate ↓ DBP ↓ SBP ↓ Blood Lactate ↑ Reaction time ↑ Dynamic balance ↑ Static balance ↔ Jump strength ↔ Manual pressure force ↔ Squat ↔ Bench press ↓ Anaerobic peak power ↓ Anaerobic minimum power ↓ Average power ↓ Fatigue index
Leonardo-Mendonça et al., 2017, Spain [52]	Randomized, double-blind, placebo-controlled, parallel group trial	24 ♂ resistance-trained athletes Age: 20.3 ± 0.71 years Weight: 74.7 ± 3.22 kg Height: 176.7 ± 1.83 cm Study withdrawals: 0 12 participants in IG 12 participants in CG	Melatonin: 100 mg per day gelatinous capsules (NR) Placebo: capsules excipients (lactose, colloidal silica) 30 min before bedtime 4 weeks	RCB Hemoglobin Hematocrit Leukocyte Glucose Total Cholesterol Triglycerides Creatinine Urea Uric Acid CK LDH AST ALT AOPP Lipid peroxidation ORAC Nitrites GSH GSSG GSH:GSSG GPx GRd GPx:GRd	↔ RBC ↔ Hemoglobin ↔ Hematocrit ↔ Leukocyte ↔ Glucose ↓ * Total Cholesterol ↔ Triglycerides ↓ Creatinine ↔ Urea ↔ Uric Acid ↓ * CK ↓ * LDH ↔ AST ↔ ALT ↓ * AOPP ↓ * Lipid peroxidation ↑ * ORAC ↓ * Nitrites ↔ GSH ↓ * GSSG ↓ * GSH:GSSG ↓ * GPx ↓ * GRd ↓ * GPx:GRd
Maldonado et al., 2012, Spain [53]	Randomized, single-blind, placebo-controlled, parallel group trial	16 ♂ professional active soccer players (from the Sevilla football club of the second division of Spain, belonging to the Spanish Professional Football League) Age (Range): 18–20 years Weight: 68.2 ± 1.5 kg Height: 177.2 ± 6.9 cm Study withdrawals: 0 8 participants in IG 8 participants in CG	Melatonin: 6 mg (NR) Placebo: (NR) 30 min before exercise 1 day	RCB Hemoglobin Hematocrit WBC Neutrophils Lymphocytes Natural Killer Ig M Ig G Ig A Cortisol Testosterone Glucose Total Cholesterol Triglycerides Total Proteins Creatinine Urea Uric Acid CK LDH AST ALT MDA TAS	↔ RCB ↔ Hemoglobin ↔ Hemoglobin ↔ WBC ↔ Neutrophils ↔ Lymphocytes ↔ Natural Killer ↔ Ig M ↔ Ig G ↑ * Ig A ↔ Cortisol ↔ Testosterone ↔ Glucose ↔ Total Cholesterol ↓ * Triglycerides ↔ Total Proteins ↔ Creatinine ↔ Urea ↔ Uric Acid ↔ CK ↔ LDH ↔ AST ↔ ALT ↓ * MDA ↑ * TAS
Mero et al., 2006, Finland [54]	Randomized, double-blind, placebo-controlled crossover trial	10 ♂ high strength and resistance-trained (regular exercise four times a week with 4.8 ± 2.0 years’ experience in strength and resistance training) Age: 24.0 ± 3.0 years Weight: 74.7 ± 5.4 kg Height: 178.0 ± 5.0 cm Body Fat: 14.3 ± 3.4% Study withdrawals: 0	Melatonin: 6 mg Tablets (University of Pharmacy, Finland), Placebo: 6 mg Tablets 60 min before exercise 1 day Washout period: 14 days	Glucose Cortisol Testosterone Grow Hormone Lactate Counter movement jump Squat Bench press Resistance Exercise Serum melatonin	↔ Glucose ↔ Cortisol ↔ Testosterone ↔ Grow Hormone ↔ Lactate ↔ Counter movement jump ↔ Squat ↔ Bench press ↔ Resistance Exercise ↑ * Serum melatonin
Ochoa et al., 2011, Spain [55]	Randomized, double-blind, placebo-controlled, parallel group trial	20 ♂ highly trained athletes daily (running) Characteristics of participants NR Study withdrawals: 0 8 participants in IG 8 participants in CG	Melatonin: 5 capsules 3 mg (Natrol, Chatsworth, CA, USA) Total dose 15 mg 1 capsule 2 days before the test with dinner, 3 capsules on the previous day (breakfast, lunch, and dinner), 1 capsule the same day of the run, 1 h before physical exercise test Placebo: beer yeast, cellulose, acacia, silica stearic acid, magnesium stearate, cellulose gum, maltodextrin 3 days	RBC Hemoglobin Reduction plasma viscosity Cholesterol Phospholipids Total Bilirubin Total Proteins Creatinine TNF-α IL-6 sTNF-α-RII IL-1ra TAS 15-F2t-Isoprostane 8-OHdG CAT GPx Serum melatonin	↔ RBC ↔ Hemoglobin ↔ Reduction plasma viscosity ↓ Cholesterol ↓ Phospholipids ↓ Total Bilirubin ↔ Total Proteins ↓ Creatinine ↓ * TNF-α ↓ * IL-6 ↑ sTNF-α-RII ↑ * IL-1ra ↑ * TAS ↓ 15-F2t-Isoprostane ↓ 8-OHdG ↑ * CAT ↑ * GPx ↑ * Serum melatonin
Ortiz-Franco et al., 2017, Spain [56]	Randomized, double-blind, placebo-controlled, parallel group trial	14 ♂ highly trained athletes Age: CG: 28.43 ± 4.39 years IG: 26.00 ± 6.03 years Weight: CG: 78.39 ± 6.68 kg IG: 79.96 ± 7.29 kg Height: CG: 176.9 ± 3.89 cm IG: 179.9 ± 6.04 cm BMI: CG: 25.06 ± 2.20 kg/m^2^ IG: 24.70 ± 19.8 kg/m^2^ Fat Mass: CG: 13.21 ± 3.91 kg IG: 14.79 ± 3.60 kg Muscle Mass: CG: 61.96 ± 4.13 kg IG: 61.94 ± 4.21 kg Study withdrawals: 0 7 participants in IG 7 participants in CG	Melatonin: 1 daily capsule containing 20 mg of melatonin (Acofarma^®^, Barcelona, Spain) Placebo: 1 daily capsule containing lactose Before exercise NR 14 days	RBC Hemoglobin Hematocrit Transferrin Ferritin Serum Iron Glucose Urea Creatinine Uric Acid Total Cholesterol HDL LDL Triglycerides Total Bilirubin Albumin Prealbumin TAC SOD GPx DNA damage Serum melatonin	↔ RBC ↔ Hemoglobin ↔ Hematocrit ↔Transferrin ↔ Ferritin ↔ Serum Iron ↔ Glucose ↔ Urea ↔ Creatinine ↔ Uric Acid ↔ Total Cholesterol ↔ HDL ↔ LDL ↔ Triglycerides ↔ Total Bilirubin ↔ Albumin ↔ Prealbumin ↑ * TAC ↔ SOD ↑ * GPx ↓ * DNA damage ↑ * Serum melatonin
Paryab et al., 2021, Tunisia [57]	Randomized, double-blind, placebo-controlled, repeated-measures crossover trial	33 ♂ university championship professional athletes (running) Age: 20.0 ± 2.0 years Weight: Body Mass: 83.4 ± 14.4 kg Height: 180.0 ± 1.0 cm Study withdrawals: 23 10 participants in IG/CG	Melatonin: 6 mg Tablets (NR) Placebo: 6 mg Tablets (NR) 30 min before training 1 day 08:00 a.m. Washout period: 3 days	Blood Lactate Reaction time Static balance Dynamic balance Anaerobic power	↓ * Blood Lactate ↓ * Reaction time ↑ * Static balance ↑ * Dynamic balance ↑ * Anaerobic power
Souissi et al., 2020 Tunisia [58]	Randomized, double-blind, placebo-controlled crossover trial	8 ♂ highly trained athletes (students of Institute of Sports and Physical Education) Age: 21.8 ± 0.9 years Weight: NR BMI: 21.0 ± 0.8 kg/m^2^ Study withdrawals: 0	Melatonin: 6 mg Capsules (NR) Placebo: 6 mg Capsules (NR) 50 min before exercise 1 day 09:00 a.m. Washout period: NR 2 test sessions at 8:00 a.m. on different days	CRP LDH AST ALT	↔ CRP ↔ LDH ↔ AST ↔ ALT

Symbols and abbreviations: ↑ = no significant increase; ↓ = no significant decrease; ↔ = no significant change. ↑ * = significant increase; ↓ * = significant decrease; *: indicates significant values (*p* < 0.05); ♂ = men. ♀ = women. 8-OHdG = 8-hydroxy-2-deoxyguanosine; ALT = alanine aminotransferase; a.m. = ante meridiem; AOPP = advanced oxidation protein products; AST = aspartate aminotransferase; BMI = body mass index; CAT = catalase; DBP = diastolic blood pressure; CG = control group; CK = creatine kinase; CRP = C-reactive protein; GPx = glutathione peroxidase; GRd = glutathione reductase; GSH = Reduced Glutathione; GSSG = oxidized glutathione; HDL = high-density lipoprotein; IG = intervention group; Ig = immunoglobulin; IL: interleukin; IL1-ra = IL-1 receptor antagonist; LDH = lactate dehydrogenase; LDL = low-density lipoprotein; MDA = malondialdehyde; min = minutes; NR = no reported; ORAC = Oxygen radical absorption capacity; ox-LDLs = oxidized low-density lipoprotein molecules; p.m. = post meridiem; RCB = red blood cells; RPE = rating of perceived exertion; SBP = systolic blood pressure; SD = standard deviation; SOD = superoxide dismutase; sTNF-α-RII = soluble receptor II of TNF-α; TAC = total antioxidant capacity; TAS = total antioxidant status; TNF-α = tumor necrosis factor-α; VAS = visual analogue scale; VO_2_max = maximum oxygen consumption; WBC = white blood cells; YYIRT-1 = Yo-Yo intermittent recovery test level 1.

## Data Availability

Not applicable.
